# Correction: Infectious diseases, comorbidities and outcomes in hospitalized people who inject drugs (PWID)

**DOI:** 10.1371/journal.pone.0303576

**Published:** 2024-05-09

**Authors:** The PLOS One Staff

## Notice of Republication

This article was republished on April 25, 2024 to correct a title error. Please download this article again to view the correct version. The publisher apologizes for the error.

## Supporting information

S1 File (PDF)

S2 File (PDF)
