# Supplementary material for: Infectious diseases, comorbidities and outcomes in hospitalized people who inject drugs (PWID)
Source: PLoS One. 2022 Apr 20;17(4):e0266663. doi: 10.1371/journal.pone.0266663 (PMC9020696; doi:10.1371/journal.pone.0266663)
Supplement: Attachment — Submitted filename: Response to Reviewers.docx [file pone.0266663.s001.docx]

Dr. E Chenette

Editor-in-Chief

PLOS ONE

Dear Dr. Chenette,

Please see below for the rebuttal letter, in which we address each of the points raised by the journal and the reviewers individually.

We wish to thank you and your team once again for reviewing our paper and providing these detailed comments on our work.

Sincerely,

Elizabeth Alvarez, MD, MPH, PhD, CMCBT

Assistant Professor, Department of Health Research Methods, Evidence and Impact (HEI)

McMaster University, Hamilton, Ontario, Canada

Journal Requirements:

When submitting your revision, we need you to address these additional requirements.

1. Please ensure that your manuscript meets PLOS ONE's style requirements, including those for file naming. The PLOS ONE style templates can be found at

<https://journals.plos.org/plosone/s/file?id=wjVg/PLOSOne_formatting_sample_main_body.pdf> and

<https://journals.plos.org/plosone/s/file?id=ba62/PLOSOne_formatting_sample_title_authors_affiliations.pdf>

**Response:** Thank you for outlining formatting requirements. We have reviewed the samples provided. Our title page and main manuscript have been revised to ensure compliance with formatting requirements as noted in the templates provided above.

2. Please include in your Methods section (or in Supplementary Information files) the participating hospitals/institutions.

**Response:** Thank you. As Reviewer #1 indicated the same suggestion, our methods section has been expanded to include a brief description of the two participating hospitals.

3. In ethics statement in the manuscript and in the online submission form, please provide additional information about the patient records/samples used in your retrospective study. Specifically, please ensure that you have discussed whether all data/samples were fully anonymized before you accessed them and/or whether the IRB or ethics committee waived the requirement for informed consent. If patients provided informed written consent to have data/samples from their medical records used in research, please include this information.

**Response:** Thank you for outlining these instructions. We have elaborated on the patient records used under our Methods section in our manuscript. We also specified that we used patient names and insurance numbers to prevent duplicate data entries. We anonymized the data set prior to statistical analyses.

4. We note that you have indicated that data from this study are available upon request. PLOS only allows data to be available upon request if there are legal or ethical restrictions on sharing data publicly. For information on unacceptable data access restrictions, please see <http://journals.plos.org/plosone/s/data-availability#loc-unacceptable-data-access-restrictions>.

In your revised cover letter, please address the following prompts:

a) If there are ethical or legal restrictions on sharing a de-identified data set, please explain them in detail (e.g., data contain potentially identifying or sensitive patient information) and who has imposed them (e.g., an ethics committee). Please also provide contact information for a data access committee, ethics committee, or other institutional body to which data requests may be sent.

b) If there are no restrictions, please upload the minimal anonymized data set necessary to replicate your study findings as either Supporting Information files or to a stable, public repository and provide us with the relevant URLs, DOIs, or accession numbers. Please see <http://www.bmj.com/content/340/bmj.c181.long> for guidelines on how to de-identify and prepare clinical data for publication. For a list of acceptable repositories, please see <http://journals.plos.org/plosone/s/data-availability#loc-recommended-repositories>.

We will update your Data Availability statement on your behalf to reflect the information you provide.

**Response:** Thank you for outlining these instructions. The team has agreed that we wish to preserve patient privacy where possible, as our data contains potentially identifying information (such as hospital sites and diagnoses) and considering the sensitivity and stigma surrounding injection drug use. Our protocol was approved by the Hamilton integrated Research Ethics Board. We have briefly summarized these sentiments and included contact information for the ethics board in our cover letter.

**Comments to the Author**

Reviewer #1: Summary: This manuscript used hospital charts/records to abstract data from people who inject drugs and hospital diagnoses and outcomes. 240 individuals were included in the analysis from two hospitals in Hamilton, Ontario. One third were admitted to the ICU, 36% had surgery, 9% died and 12% left AMA. Bacteremia was the most common diagnosis. Many of those who left AMA were seen in the ER or re-admitted within 30 days. In general, the topic of health outcomes in PWID is important to try and improve hospital care. However, the paper lacks details in some areas to help conceptualize the findings.

**Response:** Thank you for taking the time to review our manuscript, we appreciate your consideration and thoughtful feedback. We have addressed each comment to ensure our paper includes the details as suggested.

Major comments:
1. A better understanding of the hospitals included in this manuscript would be helpful—all hospitals in Hamilton? What kind of hospitals, etc. it sounds like these are urban? Those who aren’t familiar with Hamilton and/or Ontaria would benefit from a better understanding of this.

**Response:** Thank you for this suggestion, we agree that further detail on the participating hospitals would benefit readers outside of Ontario. We have therefore listed the participating hospitals and included a brief description of them in our methods section.

2. Could you please describe the data audit in more detail? What happened if discrepancies were found? Who conducted the audit?

**Response:** Thank you for your comment, we see that more explanation of the audit process is warranted to fully capture our team’s commitment to accuracy in data collection. A more thorough explanation of the data audit has been added to the methods section accordingly.

3. In general, there are many areas that I would recommend adding more detail either in the body of the text or in a supplement. What criteria were used to abstract patient data including: HIV status (based on labs, meds, diagnosis codes’]?); Hep C status (labs? Treated/untreated?); comorbid conditions? Were these included if documented in a note or need ICD9/10? A line in the discussion describes missing HIV or Hep C diagnoses if they were tested during the index admission—so was this the only way these diagnoses were captured? The index admission was used but where chart records examined prior to index admission?

**Response:** Thank you for your suggestion, we agree that the additional detail to our data extraction and collection process is needed, as the criteria for abstraction was not clear. We have elaborated upon this step in our methods section to indicate how and where data was extracted from in Meditech.

4. The use of two reviewers, a third as a tie-breaker, and an audit adds rigor to the study.

**Response:** Thank you, we appreciate this feedback. We have further expanded on our data audit process to ensure this rigor.

Minor comments:
1. Could you clarify that those charts who were excluded in paragraph 1 of the results were because they didn’t mean eligibility criteria upon further inspection?

**Response:** Thank you for suggesting this clarification, we agree this suggestion will improve the cohesiveness of our paper. We have thus restated our exclusion criteria in the first paragraph of the results section.

2. Substances used: is this inpatient? Or prior to admission? Hydromorphone is a prescription drug but is it being misused/abused/bought in these settings?

**Response:** Thank you for your comment, we agree this was unclear in our original submission. We have included a more detailed explanation of how information regarding used substances was obtained from the charts.

Reviewer #2: This manuscript is a descriptive, retrospective chart review study of PWID admitted to two hospitals with infections in a Hamilton, Ontario between 2013 and 2018. The authors report demographics, diagnoses, as well as healthcare utilization for this cohort and provide stratified results by patient-initiated and death-status. While the study nicely describes PWID/ID hospital care utilization, the manuscript lacks focus and the principal conclusions that patient directed discharges and readmissions are common have been demonstrated previously in national datasets. I think the introduction and discussion could better frame the primary hypotheses and contextualize the limited analyses performed. Additionally, the authors frame the manuscript as an initial baseline study to address longer term trajectories and improve care for PWID but do not describe any addiction interventions or withdrawal management approaches in the hospital which is essential and could be one of the strengths of retrospective chart review study like this (similar studies using publicly available databases lack detailed inpatient medication administration records or other interventions). Overall, the authors have done considerable work to gather this data yet lacks key information and requires additional focus. Below are several additional suggestions to strengthen the manuscript.

**Response:** Thank you for taking the time to review our manuscript. We greatly appreciate your insightful feedback for our paper. We would like to clarify that our goal with this chart review was the establish the feasibility of studying injection drug use in Hamilton. Studies in the past have described general statistics of people who inject drugs, but have not yet considered the nature of hospital admissions for this group. This investigation is a first step to develop a better understanding this population for future studies to be conducted surrounding trajectories and interventions.

Major comments:
1. Abstract: “little is known about outcomes” – The manuscript would be better framed with more specific identification of the gap and the goals. Which outcomes? There’s a fairly robust literature on outcomes related to specific infectious diseases (HIV, HCV, endocarditis) though less about this larger collection of diagnoses.

**Response:** Thank you for your comment, we have clarified this point in our abstract.

2. Intro: Little is known about “health trajectory of this population.” I would define this term as it is important theoretically to the framing (over what time period, what outcomes).

**Response:** Thank you for your comment, we have clarified this point in our introduction. We agree that the term “health trajectories” may not be intuitive and warrants clarification.

3. Intro: P 4, line 85: The authors argue that by including all infections among PWID together this manuscript fills a gap in the literature. Each of these infections has distinct presentations and clinical courses, I think the manuscript would be stronger with a theoretical argument about why it is crucial to group these infections together.

**Response:** Thank you for this consideration to help strengthen our paper. We have clarified the importance of considering all drug-related infections for this population accordingly.

4. P5 line 98: “This project will set the stage to further study healthcare trajectories of PWID with infections.” I’m not clear where this is going or what this means. I would suggest using this space in the manuscript instead to set up the analyses you are doing in this paper.

**Response:** Thank you for your comment, we understand our original wording may not have been clear. We have clarified our aims for our study and ensured it flows with our analysis.

5. P5 Line 99: “The overall aim…” – I would delete or make this sentence much more focused. Based on the analyses later in the manuscript, it seems the goal of this manuscript is to understand characteristics of people who do and do not have patient initiated discharges but the introduction doesn’t really set this up.

**Response:** Thank you for your suggestion, we acknowledge that this sentence is rather broad. We have modified the sentence for more specificity and incorporated the suggestion from the last comment to ensure our aims are clearer and more reflective of our work.

6. P5, Line 107 --- This is pretty well documented in the literature in addition to clinical experience.

**Response:** Thank you for your comment. We have changed this sentence to reflect this.

7. Methods: I would encourage the authors to describe a bit more about how injection drug use in the last 6 months determined/verified.

**Response:** Thank you for this excellent suggestion. Reviewer #1 expressed a similar sentiment above. As such, we have added more detail to explain our data extraction process by including how each survey query was answered and which parts of the patient chart were referenced for each query.

8. If patient-initiated discharges are indeed the focus of this paper, it would be valuable to report on medication for opioid use disorder provision in the hospital and/or withdrawal management strategy which has been shown to be linked with discharge. The authors report on medication for opioid use disorder provision prior to the hospitalization but not how the hospital approaches addiction treatment.

**Response:** Thank you for your comments. We would like to clarify that patient-initiated discharges represent one of the multiple outcomes we focused on in our investigation. Unfortunately, there is little available on how the participating hospitals respond to and address addictions treatment, aside from what was found in consult notes surrounding opioid substitution therapy. We agree that linking substitution or withdrawal management with discharge data would be an excellent addition. We hope that our study, as a first step into investigating outcomes of PWID, will be conducive to such analysis in the near future.

9. Additional analyses to consider: do people with serious deeper infections leave later in the hospitalization than people with less serious infections (cellulitis, abscesses vs endocarditis, bacteremia, etc).

**Response:** Thank you for these excellent suggestions. We agree that such analyses would yield important and insightful considerations in developing strategies to better care for this population. As mentioned above, we hope our study will allow further analyses to be carried out among this population to better understand the impact of infections of varying severities.

10. Are the comorbidities baseline or identified during the hospitalization?

**Response:** Thank you for this question. We have clarified that information on comorbidities included those at baseline (made available in the electronic records system) as well as those identified during the hospitalization.

11. Page 17 Line 293-94 – would move sentence about HIV/HCV diagnosis to the limitations section.

**Response:** Thank you for this suggestion, we agree that this makes more sense in structuring our paper. This portion originally in the fit within literature subsection has been moved above to the limitations subsection as suggested.

12. It is interesting that 55% of the population was injected unspecified opioids or hydromorphone. Can the authors provide additional information about the local context?

**Response:** Thank you for suggesting this addition. We agree that providing additional information with local data would fortify the clinical picture. We have included local data from a needs assessment and feasibility study for the Hamilton supervised injection site to help contextualize our findings. This addition was written into our discussion section, under the fit within literature subsection.

13. Limitations: This is descriptive data with bivariate analyses and does not adjust for co-variates. The authors could consider a basic, simple logistic regression model looking at factors associated with patient initiated discharge or mortality, but will be limited in the number of covariates that can be included. Regardless, the limitations section should acknowledge the limitations of this kind of analysis.

**Response:** Thank you for your comment. We agree that limitations in our statistical analyses should be recognized. We have addressed this issue in our limitations subsection.
