# Supplementary material for: Infectious diseases, comorbidities and outcomes in hospitalized people who inject drugs (PWID)
Source: PLoS One. 2022 Apr 20;17(4):e0266663. doi: 10.1371/journal.pone.0266663 (PMC9020696; doi:10.1371/journal.pone.0266663)
Supplement: Attachment — Submitted filename: Response to reviewers_2021Dec.docx [file pone.0266663.s002.docx]

Please see below responses to reviewers’ comments:

Specifically, please address reviewers' remaining concerns, and particularly specify if data underlying the findings is fully available.

RESPONSE: A data sharing and availability section was added under methods

Journal Requirements:

Please review your reference list to ensure that it is complete and correct. If you have cited papers that have been retracted, please include the rationale for doing so in the manuscript text, or remove these references and replace them with relevant current references. Any changes to the reference list should be mentioned in the rebuttal letter that accompanies your revised manuscript. If you need to cite a retracted article, indicate the article’s retracted status in the References list and also include a citation and full reference for the retraction notice.

RESPONSE: A review of referenced articles has been completed. No articles were found to have been retracted. A couple of links have been updated.

Reviewers' comments:

Reviewer's Responses to Questions

**Comments to the Author**

1. If the authors have adequately addressed your comments raised in a previous round of review and you feel that this manuscript is now acceptable for publication, you may indicate that here to bypass the “Comments to the Author” section, enter your conflict of interest statement in the “Confidential to Editor” section, and submit your "Accept" recommendation.

Reviewer #1: All comments have been addressed

Reviewer #2: All comments have been addressed

RESPONSE: Thank you

2. Is the manuscript technically sound, and do the data support the conclusions?

The manuscript must describe a technically sound piece of scientific research with data that supports the conclusions. Experiments must have been conducted rigorously, with appropriate controls, replication, and sample sizes. The conclusions must be drawn appropriately based on the data presented.

Reviewer #1: Yes

Reviewer #2: Yes

3. Has the statistical analysis been performed appropriately and rigorously?

Reviewer #1: Yes

Reviewer #2: Yes

4. Have the authors made all data underlying the findings in their manuscript fully available?

The [PLOS Data policy](http://www.plosone.org/static/policies.action#sharing) requires authors to make all data underlying the findings described in their manuscript fully available without restriction, with rare exception (please refer to the Data Availability Statement in the manuscript PDF file). The data should be provided as part of the manuscript or its supporting information, or deposited to a public repository. For example, in addition to summary statistics, the data points behind means, medians and variance measures should be available. If there are restrictions on publicly sharing data—e.g. participant privacy or use of data from a third party—those must be specified.

Reviewer #1: No

Reviewer #2: No

RESPONSE: A data sharing and availability section was added under methods

5. Is the manuscript presented in an intelligible fashion and written in standard English?

PLOS ONE does not copyedit accepted manuscripts, so the language in submitted articles must be clear, correct, and unambiguous. Any typographical or grammatical errors should be corrected at revision, so please note any specific errors here.

Reviewer #1: Yes

Reviewer #2: Yes

6. Review Comments to the Author

Please use the space provided to explain your answers to the questions above. You may also include additional comments for the author, including concerns about dual publication, research ethics, or publication ethics. (Please upload your review as an attachment if it exceeds 20,000 characters)

Reviewer #1: Thank you for responding to the reviewer comments. I think this has strengthened the manuscript. This manuscript contributes to the literature of PWID and hospitalizations, patient-initiated discharges, and deaths.

RESPONSE: Thank you!

One minor comment--hydromorphone was the most commonly injected substance, though it is also a prescription pain medication. perhaps the first time that hydromorphone is introduced in the paper that it can be described as mis-used/illicit hydromorphone? I suspect that this is hydromorphone pills that are crushed and injected as opposed to the IV hydromorphone formulation but perhaps authors can comment on this? I think there may be differences in infection risk between these two formulations...

RESPONSE: Thank you, this was clarified in the methods section under data collection.

Reviewer #2: The authors have largely addressed my comments but I think the methods section still feels a bit disjointed and does not set up the specific analyses performed. Perhaps the authors could move their discussion of sample size as it related to number of events to the statistical analysis section. The authors report detailed data collection strategy but could lay out more clearly which outcomes they will examine. The statistical analysis section can then more clearly state which comparison will be performed, etc.

RESPONSE: Clarifications were added throughout the methods section to address these points.

7. PLOS authors have the option to publish the peer review history of their article ([what does this mean?](https://journals.plos.org/plosone/s/editorial-and-peer-review-process#loc-peer-review-history)). If published, this will include your full peer review and any attached files.
If you choose “no”, your identity will remain anonymous but your review may still be made public.
**Do you want your identity to be public for this peer review?** For information about this choice, including consent withdrawal, please see our [Privacy Policy](https://www.plos.org/privacy-policy).

Reviewer #1: No

Reviewer #2: No

Thank you again for your reviews.
